# Association between Body Mass Index and Sensory Processing in Childhood: InProS Study

**DOI:** 10.3390/nu12123684

**Published:** 2020-11-29

**Authors:** Eva-María Navarrete-Muñoz, Paula Fernández-Pires, Carmela Mubarak-García, Cristina Espinosa-Sempere, Paula Peral-Gómez, Iris Juárez-Leal, Alicia Sánchez-Pérez, María-Teresa Pérez-Vázquez, Miriam Hurtado-Pomares, Desirée Valera-Gran

**Affiliations:** 1 Grupo de Investigación en Terapia Ocupacional (InTeO), Miguel Hernández University, 03550 Alicante, Spain; enavarrete@umh.es (E.-M.N.-M.); c.espinosa@umh.es (C.E.-S.); pperal@umh.es (P.P.-G.); ijuarez@umh.es (I.J.-L.); alicia.sanchez@umh.es (A.S.-P.); 2 Department of Pathology and Surgery, Miguel Hernández University, 03550 Alicante, Spain; paula.fernandezp@umh.es (P.F.-P.); cmubarak@umh.es (C.M.-G.); mt.perez@umh.es (M.-T.P.-V.); 3 Vicerrectorado de Relaciones Institucionales de la Universidad Miguel Hernández, 03202 Elche, Spain

**Keywords:** atypical movement sensitivity, atypical tactile sensitivity, body mass index, childhood obesity, sensory processing

## Abstract

We assessed the association between body mass index (BMI) and sensory processing in 445 Spanish children aged 3–7 from the InProS project. Child sensory processing was measured using the short sensory profile (SSP); an atypical sensory performance was defined as an SSP total score <155 and scores of tactile sensitivity <30; taste/smell sensitivity <15; movement sensitivity <13; under-responsive/seeks sensation <27; auditory filtering <23; low energy/weak <26; and visual/auditory sensitivity <19. The BMI was calculated according to the cutoffs by the World Health Organization for children aged 0–5 and 5–19 years. We used multiple Poisson regression models with robust variance to obtain prevalence ratios (PR). No associations between children’s overweight and obesity and the prevalence of atypical sensory outcomes were observed. A one-point increase in BMI was significantly associated with a higher prevalence of atypical tactile sensitivity (PR = 1.07, 95% CI: 1.02; 1.12). A statistically marginal association was also observed for atypical total SSP (PR = 1.03, 95% CI: 1.00; 1.07) and atypical movement sensitivity (PR = 1.05, 95% CI: 1.00; 1.10). To our knowledge, this is the first time the association between children’s BMI and sensory processing has been reported. Our findings suggest that sensory processing issues may play a part in the complex context of childhood obesity. Further research is required to confirm these findings.

## 1. Introduction

Sensory processing problems may manifest themselves in an impairment of responses to, processing of, and/or organization of sensory information. These health problems may bring about potential detrimental effects on the normal development in children by compromising their participation in functional daily life routines and activities [1,2]. Although sensory processing problems are particularly frequent among children with disabilities, the existing literature has suggested that estimates of prevalence rates for children from the general population may range from about 10% up to 55% [2,3,4,5]. Despite the fact that documented evidence for typically developing children is still notably insufficient, growing concerns about whether or not sensory processing issues may be potential factors that could adversely affect child’s health and development have opened a promising line of research based on an epidemiological approach [6].

As far as we know, several studies have suggested a likely relationship between sensory processing problems and poor dietary factors and eating behaviors during childhood [3,7,8,9,10,11]. The interest in exploring this relationship has gained considerable importance because of two main aspects: first, the process of eating involves integrating sensory domains that trigger individual sensitivity responses to food characteristics [9,12]; and second, early childhood is a critical period of life for establishing food preferences and developing sensory food aversion, which can prompt one to reject certain foods with particular tastes, textures, smells, or appearances [13]. In this regard, there is evidence that child feeding problems such as neophobia, picky eating, and/or other problematic eating behaviors have been associated with less healthy food choices that include a reduced intake of fruits, vegetables, and protein foods [7,14,15,16,17]. From the perspective of potential health consequences, it is known that an unbalanced diet during childhood can have short-term and long-term effects on children’s health and development, leading to detrimental metabolic outcomes and chronic diseases such as obesity [18,19,20,21,22,23].

Childhood obesity is one of the most serious public health challenges in the present century [22,23,24]. According to latest global estimates published by Non-Communicable Disease Risk Factor Collaboration (NCD-RisC) in 2016, the trends in the mean body mass index (BMI) and obesity prevalence in children remains at higher levels worldwide [25]. Indeed, Spain is currently among the European countries that are the most affected by the highest rates of childhood obesity, with a prevalence of 10.5% for obesity and 33.7% for overweight in children and adolescents aged 5–19 years [26]. Although childhood obesity has a multifactorial etiology resulting from an interaction of set of factors, including the environment, genetics, and ecological effects such as the family, community, and school [23], lifestyle choices such as diet, physical activity, and sedentary behaviors seem to be the main contributors to the high prevalence of this health problem [21,22,23,27]. However, the evidence is still lacking, and research findings are not fully consistent. In fact, there is currently a lack of knowledge about many factors that may potentially contribute to the development of childhood obesity. In this respect, a poor diet during childhood seems to be a health problem that has been separately associated with sensory processing problems and with childhood obesity; however, to the best of our knowledge, no previous studies have explored the relationship between these two factors. Notably, it should be noted that research findings about the association between sensory processing problems and obesity in children could provide helpful knowledge to prevent unhealthy consequences in children’s health and mitigate the onset of many chronic problems in later adult life. Hence, the present study aimed to examine the association between the body mass index and sensory processing in a population of Spanish children between the ages of 3 and 7.

## 2. Materials and Methods

### 2.1. Study Design, Participants, and Procedure

The present study is based on data from the InProS (Infancia y Procesamiento Sensorial [Childhood and Sensory Processing]) project (http://inteo.edu.umh.es/en/inpros/), a population-based cross-sectional study of preschool and school-age children between 3 and 7 years of age. Further details about the study protocol of this research project have been described elsewhere [6], and the booklet with the questionnaire used in this study is available at: http://inteo.edu.umh.es/wp-content/uploads/sites/1447/2020/01/CUESTIONARIO-final1.pdf. Briefly, the recruitment of participants was carried out during the months of February and May 2016. The study sample was chosen from a systematic random selection of 21 schools registered at the office of the Consellería de Educación, Cultura y Deporte de la Generalitat Valenciana (Education, Culture and Sport Council of the Provincial Government, http://www.ceice.gva.es), located in the Alicante province (Spain). To obtain an optimal sample of 570 children, assuming a mean of 25–30 children per school, 21 educational institutions were required for the basic premise of this study, according to which the prevalence of sensory problems in children without disabilities was 18%. Approximately 1700 eligible children were invited to participate in this study through an invitation letter addressed to their parents. The parents received an envelope containing all the information about the study: a participant information sheet outlining the project details, a booklet with several questionnaires, and the instructions on how to complete them. After a 2–3-week period, all children were asked to return the informed written parental consent and the filled-out questionnaires. A total sample of 620 children returned the required documentation, rendering a response rate of approximately 37%. For the present study, we excluded participants with missing data for outcome and exposure variables, totaling a sample of 445 (71.8%). All participants provided informed consent and had no incentive to take part in this study. Ethical approval for this research was obtained from Miguel Hernández University (DPC.ASP.02.16), and the research was performed in accordance with the Declaration of Helsinki.

### 2.2. Study Variables

#### 2.2.1. Sensory Processing

The Spanish version of the Short Sensory Profile (SSP) was used to assess children’s sensory processing [28,29]. The SSP is a screening tool designed to detect the presence of sensory processing difficulties. This tool is a parent-report questionnaire that is comprised of 38 items organized into different sections/scales according to the seven sensory systems: tactile sensitivity (i.e., tactile system), taste/smell sensitivity (i.e., olfactory and gustatory systems), movement sensitivity (i.e., vestibular and proprioception systems), under-responsive/seeks sensation (i.e., including multisensory processing), auditory filtering (i.e., hearing), low energy/weak (i.e., vestibular and proprioception systems), and visual/auditory sensitivity (i.e., visual and auditory systems). Each item is scored on a one-point to five-point scale, ranging from 1–always to 5–never. Further details about the SSP items are available in the (supplementary materials Table S1). The scores for the SSP total and SSP scales can be obtained by calculating the sum of the respective values of each item. Moreover, the SSP scoring can be used to determine children’s sensory profile (typical performance, probable difference, or definite difference) according to the cut-points proposed by Dunn [30]. Children were classified as having an atypical sensory performance according to the following scoring: SSP total score <155; and tactile sensitivity <30; taste/smell sensitivity <15; movement sensitivity <13; under-responsive/seeks sensation <27; auditory filtering <23; low energy/weak <26; and visual/auditory sensitivity <19 subscales. The internal consistency of the SSP was good (Cronbach’s α = 0.72–0.76 across scales).

#### 2.2.2. Body Mass Index

The body weight and height of children were reported by parents. Children’s BMI was calculated as the weight in kilograms divided by the square of the height in meters, and we calculated the BMI z-score according to the specific cutoffs standardized by age and sex proposed by the World Health Organization (WHO) for individuals aged 0–5 years [31] and 5–19 years [32,33]. Overweight was defined as a BMI z-score >1 standard deviation (SD), i.e., equivalent to BMI 25 kg/m^2^ at 19 years, and obesity as a BMI z-score >2 SD, i.e., equivalent to BMI 30 kg/m^2^ at 19 years.

#### 2.2.3. Other Variables

Information about children and parental sociodemographic and lifestyle factors was collected using different ad hoc questionnaires and several standardized tests that were reported by the parents [6]. In the present study, the following variables were collected: parental characteristics (age (in years), country of birth (Spain, Other), education (primary or less, secondary, university), working situation (employed, unemployed) and BMI (continuous variable)); child characteristics (age (in years), sex (boy, girl), adherence to a Mediterranean diet as measured by KIDMED index (continuous variable), sleep quantity (in hours per day), sleep quality (good, poor), TV watching (in hours per day), global physical activity (not active/moderately active; active/very active), and medical condition (yes, no)); and birth characteristics (weeks of gestation (continuous variable) and birth weight (continuous variable)). For the statistical analysis, some of these variables were categorized as follows: preterm (<37 weeks, ≥37 weeks), low birthweight (<2500 g, ≥2500 g), sleep quantity (<10, 10, >10 h per day), watching TV (≤2, >2 h per day), and adherence to a Mediterranean diet (KIDMED ≤7, >7 points).

### 2.3. Statistical Analysis

R software, version R 4.0.0 (R Core Team. R: A language and environment for statistical computing. R Foundation for Statistical Computing, Vienna, Austria; http://www.R-project.org) was used to perform the statistical analyses. All the applied statistical tests were bilateral, and the significance level was established at 0.05. The normality of the continuous variables was checked using the Kolmogorov–Sminrov test.

The sociodemographic and lifestyle characteristics of parents and their children were described according to the children’s BMI using frequencies and percentages for categorical variables, and the median and interquartile range for continuous variables due to them not being normally distributed. A Chi-square test for categorical variables and Kruskal–Wallis test for continuous variables were applied to examine differences in these characteristics by BMI. The association between the BMI as a categorical variable (i.e., normal weight, overweight, obesity) and as a continuous variable (i.e., one-point increase), and the sensory profile (i.e., typical vs. atypical performance), was assessed by multiple Poisson regression models using the cutoffs proposed for the SSP total score and the score of each SSP scale. The robust variance based on the Huber sandwich was estimated to obtain prevalence ratios (PR) and their 95% confidence interval (CI) [34,35]. Due to the log-binomial regression model not converging, a robust Poisson regression model was used instead [36]. The estimates obtained with p values between 0.05 and 0.10 were interpreted as marginally significant. Potential confounders were included in the analysis, based on factors previously identified in the literature. Moreover, the models were also adjusted for variables with p-values <0.20 in the bivariate analysis and for those whose magnitude of the effect for the exposure of interest changed by >10% following a backward elimination procedure [37]. We did not adjust for the father’s country of origin and BMI due to the large number of missing values, although we conducted a further sensitivity analysis to explore their possible influence as potential confounders. Furthermore, to assess the possible effect of the dose response in the categories of the children’s BMI, linear tests were applied for the BMI as a continuous variable (normal weight, overweight, and obesity, coded 1–3).

Several sensitivity analyses were also performed to examine the robustness of the main findings. First, the father’s country of origin and BMI (n = 383) were added to the complete model to evaluate their possible effect on the findings obtained. Second, several stratified analyses by the child’s sex and age group were performed to explore their likely influence. Moreover, based on previous evidence on the potential relationship of several conditions with children’s sensory performance or BMI, the effect of the complete model was checked after excluding children with the following features: preterm (<37 weeks of gestation; n = 50), low birthweight (<2500 g; n = 48), medical conditions (n = 37), sleeping <10 h/day (n = 120), watching TV >2 h/day (n = 187), and a low adherence to a Mediterranean diet (KIDMED ≤ 7 points; n = 216). According to the cutoffs to classify the children’s sensory profile [30], we also excluded children from the analysis who classified under probable difference and under definite difference for the SSP total and SSP scales.

## 3. Results

### 3.1. General Characteristics of Study Participants

In the present study, the prevalence of an atypical sensory performance in preschool and school-aged children was 29.4% (SSP total score <155); 11.6% (tactile sensitivity <30); 14.6% (taste/smell sensitivity <15); 21.1% (movement sensitivity <13); 47.4% (under-responsive/seeks sensation <27); 41.3% (auditory filtering <23); 12.8% (low energy/weak <26); and 27.2% (visual/auditory sensitivity <19). According to the BMI-for-age cutoffs recommended by the WHO, 68.3% of children had normal weight, 18.4% had overweight, and 13.2% were classified as having obesity.

Table 1 shows the sociodemographic and lifestyle characteristics of the participants according to the children’s BMI. As observed, few differences were found between children with normal weight, overweight, and obesity. Overall, children with obesity had a lower proportion of Spanish parents, and their mothers presented a higher median BMI and slept <10 h per day at a greater percentage than children with normal weight and/or overweight. 

### 3.2. Association between Child’s Body Mass Index and Prevalence of Atypical Sensory Performance

The results of the association between children’s BMI, assessed as a categorical and continuous variable, and the prevalence of atypical sensory processing are displayed in Table 2. The analyses of BMI categories showed no statistically significant associations with atypical sensory outcomes. However, despite the lack of statistical significance, a substantial negative effect of having a higher prevalence of tactile and movement sensitivity problems was observed for children with overweight and obesity. When assessing the associations with a one-point increase in the BMI, the negative effect of having an atypical sensory performance was evident in almost all of the SSP scales, although it was only statistically significant for atypical tactile sensitivity (PR = 1.07, 95% CI: 1.02; 1.12). In addition, we observed a statistically marginal association with having an atypical sensory performance for total SSP (PR = 1.03, 95% CI: 1.00; 1.07) and movement sensitivity (PR = 1.05, 95% CI: 1.00; 1.10).

### 3.3. Sensitivity Analyses

A set of parental and child characteristics that could be related to children’s BMI or sensory performance were examined to determine the extent to which the main results could be affected by changes in the magnitude of the effect. Table 3 presents the findings from the sensitivity analysis conducted for the associations between a one-point increase in BMI and atypical sensory outcomes in the total, tactile sensitivity, and movement sensitivity SSP scales. With a few exceptions, overall no remarkable changes were observed in the estimates found for the analyzed SSP scales. Regarding the total SSP, the magnitude of the effect substantially increased and became statistically significant when only including children aged 5 (PR = 1.10, 95% CI: 1.01; 1.20), as well as when excluding children with a low adherence to a Mediterranean diet (PR = 1.09, 95% CI: 1.01; 1.17). By contrast, the effect on the prevalence of atypical tactile sensitivity dropped considerably when excluding those children with a low Mediterranean diet (PR = 1.01, 95% CI: 0.85; 1.19). The same reduced effect on the prevalence of atypical movement sensitivity was observed when only considering children aged 3–4 (PR = 1.01, 95% CI: 0.84; 1.21), as well as when excluding children watching TV >2 h per day (PR = 1.00, 95% CI: 0.91; 1.10). However, in both cases, i.e., tactile and movement sensitivity outcomes, the associations did not reach a statistical significance. 

## 4. Discussion

This work shows that almost a third of Spanish preschool and school-age children participants in this study presented an atypical sensory performance according to the SSP total score. In parallel, it is also observed that a similar proportion of children, although slightly higher, had an excess of weight, i.e., including overweight and obesity. Despite the associations between children’s overweight and obesity and the prevalence of atypical sensory outcomes being hampered by a lack of statistical power, the main findings clearly indicated that an increase in BMI was significantly associated with a higher prevalence of atypical tactile sensitivity in children aged from 3 to 7. Moreover, after performing the sensitivity analyses, our results suggested that an increase in BMI could in all likelihood also be associated with a higher prevalence of sensory problems as measured by the total SSP score and SSP movement sensitivity scale in children in this age range. To the best of our knowledge, this is the first time the association between BMI and sensory processing has been reported in a population-based sample of children. In this regard, these estimates should raise serious concerns about factors that could affect children’s development and health, as well as serving to emphasize how little is still known about these factors. 

To date, there is no prior evidence for the association between BMI and sensory processing outcomes in children from the general population that would allow a direct comparison with other studies. On the basis that lifestyle behaviors such as a poor diet seem to play a part in children’s overweight/obesity [38] as well as in atypical sensory processing performance [8,12], a plausible explanation for our findings, however indirect, may be partly attributable to the fact that sensory processing difficulties have been linked to poor or less healthy eating behaviors in children [3,8]. Our findings about atypical tactile sensitivity are in line with those we obtained from an earlier study conducted with the same sample of Spanish children [3]. In this study, we found that children with atypical tactile sensitivity had a poorer dietary quality, measured as the adherence to a Mediterranean diet [3]. More specifically, we observed that children affected by atypical tactile sensitivity were less likely to have vegetables regularly, have cereals or grains for breakfast, and use olive oil at home [3]. These results are also consistent with previous studies that suggested that eating less fruits and vegetables was associated with tactile and taste/smell sensitivity problems [9,10,39]. In this regard, atypical tactile sensitivity as well as other sensory processing outcomes have been suggested as important contributors to food acceptance [8,12] because of sensory properties of foods such as tactile, visual, taste, and olfactory characteristics. Indeed, several studies have confirmed a close relationship between sensory processing difficulties and problematic eating behaviors [7,8,9,10,11,12]. Moreover, different studies have reported that problematic eating behaviors are related to poorer dietary choices and less balanced diets [14,15,16,17,18,19]. Importantly, the link between a poor diet and the risk of overweight/obesity has been documented by several studies conducted on Spanish children in the same age range [27,38,40]. In light of the findings, although atypical sensory processing could be seen as a proxy for a poor nutritional status, we are aware that we cannot draw a direct connection between an increase in BMI and atypical sensory outcomes due to the nature of our data. However, our results suggest that sensory processing issues should be included as a study factor that may play a part in the complex context of childhood obesity.

In our population, due to the lack of statistical power, we did not find a clear association between children’s overweight/obesity or a one-point increase in BMI and an atypical SSP movement sensitivity. However, the observed magnitude of the effect should raise concerns about the potential relationship between both factors. The SSP movement sensitivity scale can be used to identify sensory processing difficulties derived from the vestibular and proprioception systems [30]. The information obtained from these sensory systems is required for a sense of balance and spatial orientation in order to coordinate an adequate movement with balance [41]. Children with vestibular and/or proprioception dysfunctions are more prone to having difficulties in gross motor skills such as running, jumping, climbing, etc. [39], thereby tending to avoid activities that require motor coordination and balance [42,43] such as exercise or physical activity. Thus, children with such sensory conditions would be most likely to gain weight. In this regard, studies conducted in children with developmental disabilities that commonly present these sensory impairments showed that a higher BMI was associated with less participation in physical activities [44] and with a lower balance [43]. In normally developing children, several studies indicated that children with an excess of weight had a lower plantar sensitivity (which affected their motor coordination), presented a poor balance and postural control and, therefore, experienced higher difficulties in developing gross motor skills [39,42].

This study has several limitations that should be acknowledged for the interpretation of the findings. All the data collected in this study were self-reported, which could potentially result in some misclassification, although any inaccuracy in reporting should be nondifferential. In addition, the accuracy of the research data was ensured by using questionnaires that were valid and reliable instruments employed in previous research studies [6]. Regarding the sensory processing outcomes, it should be noted that SSP is not a diagnostic tool but a screening measure for identifying the presence of symptoms or diagnostic criteria. It only screens for indications that a comprehensive evaluation is needed. As such, the best professional for making an accurate diagnosis through a clinical assessment is an occupational therapist trained in sensory integration. In this respect, children classified as having a “probable difference” according to SSP scores may not necessarily have atypical sensory processing, whereby an overestimation of our findings cannot be dismissed. With respect to the BMI, another potential limitation might be that parents may misreport their child’s weight and height. However, our estimates for the BMI are very close to the rates reported by other previous studies conducted on Spanish children in the same age range [27,38,40], in which children’s weight and height were collected using standard measurements and protocols. Nevertheless, if any misclassification of the BMI occurred, it should be nondifferential. Another important limitation is directly related to the study design. In this respect, we must admit that the cross-sectional analysis of our data hinders us from establishing a causal link between a child’s BMI and the prevalence of atypical sensory processing outcomes. However, in the light of the findings obtained, this study can constitute a suitable rationale for replicating it in other larger samples by using a prospective study design. Notably, one strength of this study is that our sample was recruited from the general population and that, in order to preserve the representativeness of data, it was randomly selected. Nevertheless, we are aware that the response rate was moderately low (37%), suggesting that the yielded results must be corroborated by high-quality studies with larger samples. Moreover, it should be noted that we performed the analysis while adjusting the models for a wide range of potential confounding factors; however, the effect of unknown factors, residual confounding, or bias due to information not collected cannot be dismissed. More importantly, since many statistical tests were applied in this study, we must recognize the problem of multiple testing, which involves the fact that our results could be due to the likelihood of a chance finding. As such, the significant association that was observed between an increase in BMI and a higher prevalence of atypical tactile sensitivity should be interpreted with caution and confirmed by further population-based prospective studies. Finally, we conducted sensitivity analyses to check the robustness of the findings, considering specific conditions that could interfere or be related to the children’s BMI and sensory processing outcomes.

## 5. Conclusions

This is the first time that a population-based study provides epidemiological evidence of the potential association between overweight/obesity and the prevalence of an atypical sensory performance in children between the ages of 3 and 7. Our estimates confirm that childhood obesity remains a crucial public health challenge, although they also show that sensory processing issues may be a health concern affecting the lives of a considerable proportion of children from the general population. Despite the lack of statistical power, our findings suggest that a greater BMI may be associated with a higher prevalence of atypical tactile sensitivity. Moreover, in all probability, a higher BMI could be related to a higher presence of sensory problems, as measured by the total SSP score and SSP movement sensitivity scale in children in this age range. This study highlights that research on childhood obesity should include information about potential factors, such as sensory processing issues, that could tend to coexist and/or interrelate with other factors affecting children’s health. Further prospective research should corroborate our results in order to help develop interventions that tackle the determinants of childhood obesity within a wider complex health context.

## Figures and Tables

**Table 1:** Demographic and lifestyle characteristics according to the child body mass index categories of the participants in the InProS Project (*n* = 445).

	Total	Body Mass Index ^1^			
		Normal Weight (n = 304)	Overweight (n = 82)	Obesity (n = 59)	*p* ^3^
**Maternal characteristics**					
Age (years), median (IR)	38 (35; 41)	38 (35; 42)	38 (35; 41)	37 (35; 40)	0.369
Country of birth (Spanish), %	85.6	88.2	82.9	76.3	0.044
Education (University studies), %	44.5	46.1	46.3	33.9	0.269
Working situation (yes), %	70.6	73.0	65.9	64.4	0.241
BMI, median (IR)	22.8 (20.7; 25.4)	22.5 (20.4; 25.0)	22.7 (21.0; 25.7)	24.5 (21.6; 26.5)	0.010
**Paternal characteristics ^2^**					
Age (years), median (IR)	39.0 (37.0; 43.0)	39.0 (37.0; 43.0)	40.0 (37.5; 43.0)	40.0 (37.5; 42.0)	0.584
Country of birth (Spanish), %	84.3	86.4	85.1	72.5	0.050
Education (University studies), %	35.5	35.8	37.3	31.4	0.429
Working situation (yes), %	90.3	91.7	88.1	86.3	0.382
BMI, median (IR)	25.6 (23.8; 27.7)	25.6 (23.5; 27.5)	25.3 (24.3; 27.7)	25.9 (24.3; 29.2)	0.346
Child characteristics					
Age (years), median (IR)	5 (4; 6)	5 (4; 6)	5 (4; 6)	6 (5; 6)	0.581
Sex (female), %	47.6	49.3	48.8	37.3	0.231
Adherence to MD, median (IR)	8 (6; 9)	8 (7; 9)	7 (6; 9)	7 (6; 9)	0.473
Sleep (<10h/day), %	27.0	25.0	24.4	40.7	0.013
Sleep quality (poor); %	9.4	9.5	7.3	11.9	0.656
TV (h/day), median (IR)	2.0 (1.3; 2.6)	2.0 (1.3; 2.6)	1.9 (1.3; 2.3)	2.3 (1.4; 2.6)	0.210
Physical activity (very active/active), %	59.9	58.9	57.3	67.8	0.389

IR: Interquartile range; MD, Mediterranean diet; ^1^ The categories of the body mass index were obtained using the cutoffs proposed by the World Health Organization, adjusted by age and sex; ^2^ Paternal information is available for 383 parents; ^3^ P-value from the Chi-square test (categorical variables) and Kruskall–Wallis (continuous variables).

**Table 2:** Association between the body mass index (as a categorical and continuous variable) and the prevalence of atypical sensory processing using the total and subscales scores of SSP in children aged 3–7 years from InProS Project (*n* = 445).

	Normal Weight (n = 304)	Overweight (n = 82)			Obesity (n = 59)			P-Trend ^1^	One-Point Increase		
Atypical Sensory Performance	n	n	PR ^2^ (95% CI)	P	N	PR ^2^ (95% CI)	P		n	PR ^2^ (95% CI)	*p*
SSP total score (<155 points)	90	21	0.84 (0.56; 1.27)	0.415	20	1.01 (0.68; 1.48)	0.974	0.815	131	1.03 (1.00; 1.07)	0.072
Tactile sensitivity (<30 points)	29	13	1.52 (0.82; 2.84)	0.187	10	1.40 (0.70; 2.81)	0.340	0.528	52	1.07 (1.02; 1.12)	0.004
Taste/smell sensitivity (<15 points)	47	9	0.66 (0.34; 1.29)	0.228	9	0.81 (0.43; 1.52)	0.517	0.575	65	1.02 (0.95; 1.09)	0.666
Movement sensitivity (<13 points)	58	20	1.20 (0.76; 1.88)	0.433	16	1.27 (0.79; 2.04)	0.330	0.276	94	1.05 (1.00; 1.10)	0.073
Under-responsive/seeks sensation (<26 points)	141	37	0.96 (0.73; 1.26)	0.775	33	1.12 (0.86; 1.46)	0.398	0.698	211	1.02 (0.99; 1.05)	0.267
Auditory filtering (<23 points)	127	30	0.86 (0.63; 1.19)	0.372	27	1.02 (0.75; 1.39)	0.907	0.863	184	1.02 (0.98; 1.05)	0.366
Low energy/weak (<26 points)	42	6	0.52 (0.22; 1.23)	0.134	9	0.93 (0.50; 1.73)	0.812	0.756	57	1.01 (0.93; 1.09)	0.795
Visual/auditory sensitivity (<19 points)	84	22	0.93 (0.62; 1.40)	0.742	15	0.79 (0.51; 1.23)	0.294	0.436	121	0.99 (0.95; 1.04)	0.742

CI: Confidence Interval; SSP: Short sensory profile; ^1^ To calculate the P-trend, the values 0, 1, and 2 were assigned to the normal weight, overweight, and obesity categories of the body mass index in order to enter the variable into the model as a continuous term. ^2^ PR: Prevalence Ratio adjusted for children’s sleep quantity (<10; 10; >10 h/day), mother’s country of birth (Spain; other country), and mother’s body mass index.

**Table 3:** Sensitivity analysis of the association between the body mass index (in categories and continuously) and the prevalence of atypical tactile sensitivity as measured by the SSP in children aged 3 to 7 years from InProS Project, Alicante, Spain (*n* = 445).

	Body Mass Index (One-Point Increase)								
	SSP Total Score (<155 Points)			Tactile Sensitivity (<30 Points)			Movement Sensitivity (<13 Points)		
	Cases/Total	PR (95% CI)	P	Cases/Total	PR (95% CI)	P	Cases/Total	PR (95% CI)	*p*
Complete model ^1^	131/445	1.03 (1.00; 1.07)	0.072	52/445	1.07 (1.02; 1.12)	0.004	94/445	1.05 (1.00; 1.10)	0.073
Adjusted by father’s body mass index	110/383	1.03 (0.99; 1.07)	0.149	45/383	1.06 (1.01; 1.12)	0.031	80/383	1.06 (1.00; 1.12)	0.035
Only boys	83/233	1.03 (0.99; 1.07)	0.157	33/233	1.06 (1.01; 1.12)	0.017	56/233	1.03 (0.97; 1.10)	0.353
Only girls	48/212	1.04 (0.97; 1.12)	0.263	19/212	1.06 (0.94; 1.19)	0.345	38/212	1.06 (0.97; 1.17)	0.214
Only children aged 3–4	48/134	1.02 (0.93; 1.12)	0.663	23/134	1.09 (0.92; 1.28)	0.311	26/134	1.01 (0.84; 1.21)	0.940
Only children aged 5	42/151	1.10 (1.01; 1.20)	0.030	17/151	1.08 (0.95; 1.24)	0.247	31/151	1.05 (0.95; 1.16)	0.366
Only children aged 6–7	41/161	1.03 (0.98; 1.08)	0.317	12/161	1.09 (1.03; 1.17)	0.007	37/161	1.05 (0.98; 1.12)	0.202
Excluding preterm	102/356	1.04 (0.99; 1.09)	0.090	44/356	1.06 (0.98; 1.15)	0.168	76/356	1.08 (1.02; 1.14)	0.013
Excluding low birthweight	112/382	1.04 (0.99; 1.09)	0.097	45/382	1.08 (1.00; 1.17)	0.049	84/382	1.06 (1.00; 1.13)	0.038
Excluding children with some medical conditions	115/407	1.04 (1.00; 1.07)	0.043	48/407	1.08 (1.02; 1.13)	0.005	83/407	1.05 (0.99; 1.11)	0.087
Excluding children sleeping <10 h/day	91/325	1.03 (0.96; 1.10)	0.432	37/325	1.07 (0.96; 1.18)	0.221	68/325	1.03 (0.96; 1.11)	0.391
Excluding children watching TV >2 h/day	63/258	1.03 (0.95; 1.12)	0.517	23/258	1.05 (0.93; 1.18)	0.465	43/258	1.00 (0.91; 1.10)	0.999
Excluding children with low adherence to MD	56/224	1.09 (1.01; 1.17)	0.020	17/224	1.01 (0.85; 1.19)	0.937	41/224	1.06 (0.97; 1.17)	0.200
Excluding children with probable atypical SP	55/369	1.03 (0.99; 1.07)	0.145	27/420	1.08 (1.02; 1.15)	0.008	46/397	1.08 (1.01; 1.15)	0.024
Excluding children with definite atypical SP	76/390	1.04 (0.97; 1.12)	0.299	25/418	1.06 (0.95; 1.17)	0.319	48/399	1.02 (0.94; 1.10)	0.679

Abbreviations: PR, Prevalence Ratio; CI, Confidence Interval; SSP, Short sensory profile; MD, Mediterranean Diet; SP, sensory processing. ^1^ Model adjusted for child’s sleep quantity (<10; 10; >10 h/day), mother’s country of birth (Spain; other country), and body mass index.

## Supplementary Materials

The following are available online at https://www.mdpi.com/2072-6643/12/12/3684/s1, Table S1: Summary of the Short Sensory Profile items.
