# Supplementary material for: Association between Body Mass Index and Sensory Processing in Childhood: InProS Study
Source: Nutrients. 2020 Nov 29;12(12):3684. doi: 10.3390/nu12123684 (PMC7761512; doi:10.3390/nu12123684)
Supplement: Supplementary file 1 [file nutrients-12-03684-s001.pdf]

Table S1. Summary of the Short Sensory Profile items*

Item	Tactile sensitivity
1	Expresses distress during grooming (for example. fights or cries during haircutting, face washing, fingernail cutting)
2	Prefers long-sleeved clothing when it is warm or short sleeves when it is cold
3	Avoids going barefoot, especially in sand or grass
4	Reacts emotionally or aggressively to touch
5	Withdraws from splashing water
6	Has difficulty standing in line or close to other people
7	Rubs or scratches out a spot that been touched
**Item**	**Taste/smell sensitivity**
8	Avoids certain tastes of food smells that are typically part of children's diets
9	Will only eat certain tastes
10	Limits self to particular food textures/temperatures
11	Picky eater, especially regarding food textures
**Item**	**Movement sensitivity**
12	Becomes anxious or distressed when feet leave the ground
13	Fears falling or heights
14	Dislikes activities where head is upside down (for example, somersaults, roughhousing)
**Item**	**Underresponsive/seeks sensation**
15	Enjoys strange noises/seeks to make noise for noise's sake
16	Seeks all kinds of movement and this interferes with daily routines (for example, can't sit still, fidgets)
17	Becomes overly excitable during movement activity
18	Touches people and objects
19	Doesn't seem to notice when face or hands are messy
20	Jumps from one activity to another so that it interferes with play
21	Leaves clothing twisted on body
**Item**	**Auditory Filtering**
22	Is distracted or has trouble functioning if there is a lot of noise around
23	Appears to not hear what you say (for example, does not "tune-in" to what you say, appears to ignore you)
24	Can't work with background noise (for example, fan, refrigerator)
25	Has trouble completing tasks when the radio is on
26	Doesn't respond when name is called but you know the child's hearing is OK
27	Has difficulty paying attention
**Item**	**Low energy/weak**
28	Seems to have weak muscles
29	Tires easily, especially when standing or holding particular body position
30	Has a weak grasp
31	Can't lift heavy objects (for example, weak in comparison to same age children)
32	Props to support self (even during activity)
33	Poor endurance/tires easily
**Item**	**Visual/auditory sensitivity**
34	Responds negatively to unexpected or loud noises (for example, cries or hides at noise from vacuum cleaner, dog barking, hair dryer)
35	Holds hands over ears to protect ears from sound
36	Is bothered by bright lights after others have adapted to the light

37	Watches everyone when they move around the room
38	Covers eyes or squints to protect eyes from light

*Rating scale ranged from 1 to 5: 1=always; 2=frequently; 3=occasionally; 4=seldom; 5=never.
